# Empowering Patients, Preventing Strokes: Knowledge of Risk Factors and Associated Factors, Prevention Strategies, and Treatment Awareness Among Chronic Disease Patients in Southern Ethiopia

**DOI:** 10.1155/bn/7202918

**Published:** 2026-03-28

**Authors:** Takla Tamir, Selamawit Yizelkal, Bekahegn Girma, Abraham Dessie Gessesse, Atrsaw Shimekaw

**Affiliations:** ^1^ Department of Nursing, College of Medicine and Health Science, Dilla University, Dilla, Ethiopia, du.edu.et; ^2^ Department of Internal Medicine, College of Medicine and Health Science, Dilla University, Dilla, Ethiopia, du.edu.et

**Keywords:** factors, knowledge, prevention, risk factors, stroke, treatment awareness

## Abstract

**Background:**

The burden of stroke is largely due to modifiable risk factors. Lack of knowledge about stroke and its risk factors hinders prevention and treatment. Effective health promotion programs need context‐specific data on the target population′s knowledge and practices. However, there is a shortage of data on the public′s understanding of stroke and its risk factors in sub‐Saharan Africa.

**Objective:**

This study is aimed at assessing the knowledge of chronic disease patients about stroke risk factors, prevention methods, treatment options, and associated factors.

**Methods:**

This cross‐sectional study was conducted in southern Ethiopia from February to April 2024. A total of 430 chronic disease patients were included by systematic random sampling. Data were collected using a structured questionnaire and medical records. Statistical analysis was performed using SPSS. Ethical clearance was obtained.

**Result:**

A total of 430 patients participated, with hypertension being the most prevalent chronic disease. Only 25.1% demonstrated good knowledge of stroke risk factors.

In multivariable analysis, good knowledge of stroke risk factors was significantly associated with receiving care in private institutions (AOR = 2.6; 95% CI: 1.1–5.9), having a degree or higher education (AOR = 6.5; 95% CI: 1.9–21.9), urban residence (AOR = 3.3; 95% CI: 1.4–7.9), family history of stroke (AOR = 5.3; 95% CI: 2.1–13.0), appropriate follow‐up (AOR = 3.4; 95% CI: 1.4–8.4), and prior information from healthcare providers (AOR = 2.2; 95% CI: 1.0–4.5). In contrast, merchants, farmers/housewives, and other occupational groups had significantly lower odds of good knowledge compared with employees. Some estimates showed wide confidence intervals, indicating limited precision and the need for cautious interpretation. Awareness of treatment and prevention methods was limited, with only 33.3% answering over 50% of prevention questions correctly.

**Conclusion:**

The study revealed a significant lack of knowledge about stroke risk factors and prevention methods among chronic disease patients in southern Ethiopia. This highlights the urgent need for targeted health education interventions. Implementing comprehensive health promotion programs that address stroke‐related knowledge gaps is crucial. These should be tailored to specific demographics and delivered through various channels, healthcare settings, communities, and media.

## 1. Introduction

Stroke mortality and disability can be reduced in low‐income countries by addressing key risk factors: hypertension, high cholesterol, diabetes, and unhealthy lifestyle habits (e.g., smoking, inactivity, poor diet, and obesity) [1].

Most strokes are preventable by managing blood pressure, eating healthy, exercising regularly, and quitting smoking. A study found that these five factors contribute significantly to stroke risk: blood pressure, diet, inactivity, smoking, and abdominal obesity, which accounted for 82% and 90% of the population‐attributable risk (PAR) for ischemic and hemorrhagic stroke, respectively [2].

Similarly, the Global Burden of Disease Study revealed that modifiable risk factors were responsible for 90.5% of the global burden of stroke [3]. The leading cause of stroke risk factors in Ethiopia was hypertension, which accounted for 50.9% of all cases. Cardiovascular disorders (16.6%), diabetes mellitus (DM) (7.4%), alcohol (10.4%), cigarette smoking (4.9%), and TB meningitis (3.1%) were the next leading causes. Mortality from stroke in hospitals was 14.7% [4].

Lack of knowledge about stroke and its risk factors in the population is a major obstacle to prevention and treatment. Effective health promotion programs require context‐specific data about the target population′s knowledge and practices. However, there is a shortage of data on the public′s understanding of stroke and its risk factors in sub‐Saharan Africa [5].

Stroke is a major global health problem. In 2019, there were 101.5 million stroke cases worldwide, resulting in 6.6 million deaths. Ischemic stroke accounted for 3.3 million deaths, intracerebral hemorrhage for 2.9 million, and subarachnoid hemorrhage for 0.4 million [6, 7]. Every 2 s, someone has a stroke, and every 6 s, someone dies from a stroke [8]. Stroke is the second leading cause of death and the third leading cause of disability globally. More than three‐quarters of strokes occur in low‐ and middle‐income countries, and stroke incidence has doubled in these countries over the past 40 years [1, 9].

Despite knowing how to prevent stroke through modifiable risk factors, it remains a major global health problem [10]. Hypertension is a significant risk factor, increasing stroke risk by fourfold [11]. In Ethiopia, over half of stroke risk factors are related to hypertension, making it the most prevalent modifiable risk factor, followed by cardiac disease and DM [12]. About 80% of people with high blood pressure experience stroke [13].

Research showed that there were differences in patient understanding of stroke prevention between nations. For instance, an Indian study on hypertensive patients′ understanding of stroke prevention found that 70% of them had insufficient information, whereas 30% had moderate knowledge [14].

In Nigeria, a study among hypertensive patients on knowledge and practices of stroke prevention showed that 90.8% of the participants had good knowledge of stroke prevention modalities [15]. A recent study conducted in Debre Tabor revealed that only 24.9% of participants had good knowledge of stroke prevention methods [16].

According to the World Health Organization report (2017) in Ethiopia, stroke was the second leading cause of death (6.23%), and it was a massive financial burden not only for patients but also for society as a whole. Therefore, public stroke knowledge will be the key tip in stroke prevention activities [17]. It is a preventable disease, with prevention strategies mainly based on knowledge about stroke risk factors [18, 19].

Furthermore, people with hypertension, cardiac disease, and DM are at high risk for developing a stroke [4, 20]. Therefore, to prevent the occurrence of stroke, assessing the knowledge of risk factors and prevention methods of stroke among chronic disease patients is very important. There is a scarcity of information on chronic disease patients′ knowledge of stroke risk factors, and it is also affected by sociodemographic attributes across the country [21]. Hence, this study is aimed at assessing the knowledge of chronic disease patients on risk factors and prevention methods, treatment awareness of stroke, and its associated factors at healthcare settings in southern Ethiopia.

The findings will help healthcare settings in southern Ethiopia plan and implement interventions to improve stroke prevention and treatment. By providing health education on stroke risk factors and prevention methods, healthcare providers can reduce the burden on patients and their families. This study will also serve as a baseline for future research on stroke prevention in the region.

## 2. Methods and Materials

### 2.1. Study Design Area and Period

A cross‐sectional study was conducted at selected healthcare settings of Gedeo Zone in southern Ethiopia. There was one general hospital, four primary hospitals (one is private), 38 governmental health centers, and 24 medium private clinics (one specialty clinic providing chronic follow‐up services) in the area. Data were collected from patients and their medical records during routine follow‐up visits from February 5 to April 26, 2024. Patient interviews were conducted, followed by a review of their medical records on the same day.

### 2.2. Population

The source population consisted of all chronic disease patients who followed up at Gedeo Zone healthcare settings. The study population included all chronic disease patients who visited the chronic follow‐up clinic during the data collection period.

### 2.3. Eligibility Criteria

All chronic disease patients who visited the chronic follow‐up clinic in the Gedeo Zone healthcare settings during the data collection period and had at least one prior follow‐up were included in the study. Patients who were seriously ill or unable to communicate, as well as those who had already developed a stroke prior to the data collection period, were excluded.

### 2.4. Sample Size Determination

The sample size was calculated using the StatCalc function of Epi‐Info Version 7. The single population proportion formula was used for knowledge, and the double population proportion formula was used for associated factors. The maximum sample size calculated for all objectives was 396. Adding a 10% nonresponse rate increased the total sample size to 436.

### 2.5. Sampling Methods and Procedures

The study was conducted in selected healthcare settings of the Gedeo Zone, southern Ethiopia. The sampling frame comprised all health facilities in the zone; however, only facilities providing structured chronic disease follow‐up services were eligible for inclusion. Accordingly, a total of 12 healthcare settings (e.g., public and private hospitals, health centers, and clinics) were selected to ensure representation across different service levels and ownership types.

Prior to participant recruitment, a 3‐month retrospective review of chronic disease follow‐up records was conducted at each selected facility to determine the average monthly patient flow. This assessment revealed differences in patient volume across facilities, while the distribution of major chronic conditions (hypertension, DM, and cardiac diseases) was generally comparable.

The total sample size was allocated proportionally to each healthcare setting based on the average monthly patient volume using probability proportional to size (PPS) sampling. Within each facility, eligible participants were selected using systematic random sampling. The sampling interval was determined by dividing the estimated monthly patient flow by the allocated sample size for each facility, resulting in a sampling interval of two (*K* = 2). Accordingly, every second eligible patient attending the chronic disease follow‐up clinic during the data collection period was selected for interview.

To minimize potential periodicity bias, the first participant was selected using a random starting point at each facility and on each data collection day. In addition, variations in appointment schedules and patient arrival patterns across facilities reduced the likelihood of systematic ordering of patients. When irregular patient flow patterns were observed, data collectors were instructed to select the next eligible patient to maintain randomization. Although variations in appointment scheduling may reduce systematic ordering effects, periodicity bias cannot be entirely excluded.

### 2.6. Data Collection Techniques and Tools

Data were collected using a structured, closed‐ended Stroke Recognition Questionnaire (SRQ), adapted from previously validated instruments reported by Arisegi et al. [15] and Tibebu et al. [16]. The questionnaire was designed to assess participants′ knowledge of stroke risk factors, prevention methods, and treatment options. It consisted of multiple sections with a total of 48 items, primarily formatted as dichotomous (yes/no/I do not know) and multiple‐choice questions. Content validity was established through expert review by two subject‐matter specialists (an internist and a senior public health researcher), who evaluated the tool for relevance, clarity, and cultural appropriateness. Based on their feedback, minor modifications were made, including rephrasing of medical terminology and contextual adaptation to ensure comprehension by the local population.

The questionnaire was pretested on 5% of the total sample in a nonselected healthcare facility prior to the main data collection. Internal consistency reliability was assessed using Cronbach′s alpha. The knowledge subscales (risk factors and prevention methods) demonstrated acceptable reliability, with Cronbach′s alpha coefficient values of 0.73 and 0.82, respectively.

Data were collected through face‐to‐face, interviewer‐administered interviews conducted by trained data collectors. To complement self‐reported information, participants′ clinical profiles were extracted from medical records on the same day as the interview. Data collection was conducted from February 5 to April 26, 2024. The complete questionnaire has been provided as supplementary material to enhance transparency and reproducibility.

### 2.7. Operational Definition

#### 2.7.1. Chronic Disease

Chronic diseases include hypertension, cardiac disease, and DM.

#### 2.7.2. Knowledge

Knowledge of stroke risk factors and prevention methods was assessed using composite scores derived from dichotomous (correct/incorrect) items. In the absence of a universally validated cutoff for stroke knowledge scales, participants who correctly answered ≥ 50% of the total items were categorized as having “good knowledge” [15, 16]. This threshold was selected primarily to ensure comparability with prior community‐based studies conducted in similar low‐ and middle‐income settings, where a 50% midpoint has been commonly applied to distinguish relatively adequate from inadequate knowledge levels. We acknowledge that a 50% cutoff does not represent a psychometrically established standard of adequacy and should therefore be interpreted as a pragmatic classification rather than a definitive benchmark of sufficient knowledge.

### 2.8. Data Quality Control Measures

Maximum effort was invested to ensure data quality at all stages. A well‐designed questionnaire was translated into the local language and back‐translated into English to ensure consistency. A pretest was done from May 21 to 30, 2023. Data collectors and supervisors were trained. Checking, coding, and cleaning of data were done before analysis.

### 2.9. Method of Data Processing and Analysis

Data were exported to SPSS for analysis. Frequency, percentage, median, and standard deviations were used to describe the study population, and the results were presented in text, tables, and figures. In addition to categorical classification (≥ 50% vs. < 50%), continuous knowledge scores were examined descriptively to characterize the distribution of responses. Mean scores, standard deviations, and observed ranges were calculated to assess central tendency and dispersion prior to dichotomization. Binary categorization was retained for regression analysis to enhance interpretability and allow comparability with similar studies conducted in comparable settings. However, continuous score distribution was evaluated to ensure that the chosen cutoff did not obscure substantial clustering or distributional irregularities.

To assess overall knowledge of stroke prevention strategies, a binary scoring system was employed where each correct identification received one point. Knowledge items were equally weighted (one point per correct response), as the instrument is aimed at assessing recognition rather than the relative clinical impact of individual prevention strategies. Given variability in reported effect sizes across populations and study contexts, deriving standardized weights was not feasible without introducing additional assumptions. Equal weighting ensured transparency and comparability with similar cross‐sectional studies.

Independent variables with *p* < 0.25 in bivariable logistic regression were entered into the multivariable model using the purposeful selection approach to control for potential confounding. Although variables with *p* < 0.25 in bivariable analysis were initially considered for multivariable logistic regression based on the purposeful selection strategy, we re‐evaluated the bivariable results using a more conservative threshold of *p* < 0.20. The same variables met the inclusion criteria at both thresholds, indicating that the final model was robust and not affected by the choice of cutoff. Multicollinearity was assessed using VIF values and standard errors; all VIFs were < 2.5, indicating that multicollinearity was not a concern. Model fitness was evaluated using the Hosmer–Lemeshow test (*p* = 0.741), indicating good fit. The Omnibus Tests of Model Coefficients were significant (*p* < 0.001), confirming that the final model improved prediction compared to the null model. A *p* value of < 0.05 is declared as statistically significant.

### 2.10. Ethics Statement

Ethical clearance was obtained from the IRB of Dilla University (duirb049/23‐07). Prior approval was secured from each participating hospital. Written, informed, voluntary, and signed consent was obtained from each participant. In accordance with the Declaration of Helsinki, all participant information was kept confidential, and data were analyzed anonymously.

## 3. Result

### 3.1. Sociodemographic Characteristics

A total of 430 clients participated in this study, resulting in a high response rate of 98.6%. The majority of participants (88.6%) received services in governmental healthcare settings. Additionally, over half (53.5%) of the participants belonged to the 41–60‐year age group (Table 1).

**Table 1:** Sociodemographic characteristics of chronic disease patients in southern Ethiopia.

Variables		Frequency (*n*)	Percentage (%)
Type of institution the participant served	Governmental	381	88.6
	Private	49	11.4
Age	20–40	104	24.2
	41–60	230	53.5
	> 60	96	22.3
Sex	Male	219	50.9
	Female	211	49.1
Residence	Urban	261	60.7
	Rural	169	39.3
Marital status	Single	34	7.9
	Married	350	81.4
	Divorced and widowed	46	10.7
Occupation	Employee	77	17.9
	Merchant	102	23.7
	Farmer	80	18.6
	Housewife	136	31.6
	Labor worker, retired, and student	35	8.2

### 3.2. Clinical Profiles

Hypertension was most prevalent, affecting 66% of participants. Additionally, 28% have DM, and 7.7% have cardiac disease. Notably, nearly 3% of participants suffer from all three chronic conditions simultaneously. Furthermore, 8% of participants have a family history of stroke (Table 2).

**Table 2:** Clinical profiles of chronic disease patients in southern Ethiopia.

Variables		Frequency	Percentage
Hypertension (HTN)	Yes	284	66.0
	No	146	34.0
Diabetes mellitus (DM)	Yes	226	52.6
	No	204	47.4
Cardiac disease	Yes	80	18.6
	No	350	81.4
Hypertension & diabetes mellitus	Yes	302	70.2
	No	128	29.8
Hypertension & cardiac disease	Yes	397	92.3
	No	33	7.7
Diabetes mellitus & cardiac disease	Yes	414	96.3
	No	16	3.7
Hypertension, diabetes mellitus, & cardiac disease	Yes	418	97.2
	No	12	2.8
Additional disease	Yes	13	3.0
	No	417	97.0
Family history of stroke	Yes	36	8.4
	No	394	91.6
Having an appropriate follow‐up	Yes	317	73.7
	No	113	26.3

### 3.3. Information‐Related Variables

The study found that only 21% of participants reported receiving information about stroke directly from their healthcare providers. However, a significant proportion of participants (29%) had personal experience with stroke through someone they knew, and almost 49% had exposure to stroke information from various sources, with media being the most common (Table 3).

**Table 3:** Information about stroke among chronic disease patients in southern Ethiopia.

Variables		Frequency	Percentage
Did the healthcare providers tell you about a stroke	Yes	91	21.2
	No	339	78.8
Do you know someone with a stroke	Yes	124	28.8
	No	306	71.2
Have you heard about a stroke	Yes	205	47.7
	No	225	52.3
Source of information (*n* = 205)	Health professionals	93	45.4
	Media (print/electronic)	110	53.7
	Read about stroke	23	11.2
	Discussion with family/friends	21	10.2
	Seen someone who had a stroke	112	54.6
	Taught in school	10	4.9
	Other	4	1.9
Most contacted profession	Specialist	64	14.9
	General practitioner	205	47.7
	Nurse	138	32.1
	Health officer	23	5.3

*Note:* Participants could select more than one source of information, so percentages may sum to more than 100% (*n* = 205 responses).

### 3.4. Knowledge About Risk Factors of Stroke

Only 42.6% correctly identified high blood pressure as a risk factor, and fewer than half recognized the dangers of DM, heart disease, and an unhealthy diet (Table 4).

**Table 4:** Knowledge on risk factors of stroke among chronic disease patients in southern Ethiopia.

Question	Response	*N*	%
High blood pressure is a risk factor for stroke	Yes	183	42.6
	No	247	57.4
Diabetes mellitus is a risk factor for stroke	Yes	134	31.2
	No	296	68.8
Heart disease is a risk factor for stroke	Yes	137	31.9
	No	293	68.1
Abnormal blood cholesterol level is a risk factor for stroke	Yes	121	28.1
	No	309	71.9
An unhealthy diet/excess fat in the diet is a risk factor for stroke	Yes	123	28.6
	No	307	71.4
Smoking is a risk factor for stroke	Yes	109	25.3
	No	321	74.7
Obesity is a risk factor for stroke	Yes	124	28.8
	No	306	71.2
Drinking excessive alcohol is a risk factor for stroke	Yes	127	29.5
	No	303	70.5

The mean score for stroke risk factor knowledge was 2.06 (SD = 1.60), with observed scores ranging from 0 to 8. Based on the predefined 50% cutoff, 25.1% of participants were categorized as having good knowledge; the distribution of scores did not demonstrate evidence of bimodality. A larger proportion of participants scored below the 50% threshold, indicating a right‐skewed distribution consistent with generally low knowledge levels. No significant ceiling effects were observed, while mild floor clustering was noted among participants with minimal recognition of risk factors.

**Figure 1:**
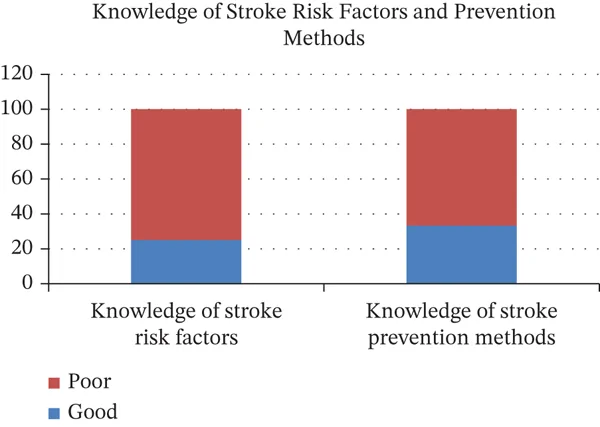
Knowledge of stroke risk factors and prevention methods among chronic disease patients in southern Ethiopia.

Overall, only 108 out of 430 participants (25.1%, confidence interval: 21–30) scored above 50% correct answers on the stroke risk factor questions and were categorized as having good knowledge (Figure 1).

### 3.5. Factors Associated With Knowledge of Risk Factors of Stroke

The study identified several factors associated with participants′ knowledge about stroke risk factors. These included the type of institution, age, sex, educational status, residence, occupation, duration of follow‐up, hypertension, family history, appropriate follow‐up, and being informed by healthcare providers.

A multivariate logistic regression analysis, with an Omnibus Test of Model Coefficients value of 0.000 and a Hosmer–Lemeshow test value of 0.741, revealed that type of institution, educational status, residence, occupation, family history, appropriate follow‐up, and being informed by healthcare providers were independently associated with knowledge about stroke risk factors.

Participants who served in private institutions were 2.6 times more likely to have good knowledge compared to participants who served in governmental hospitals (1.1 and 5.9). Participants who had a degree and above educational status were 6.5 times more likely to have good knowledge compared to participants who had no formal education (1.9 and 21.9). Participants living in urban areas were 3.3 times more likely to have good knowledge compared to participants living in rural areas (1.4 and 7.9). On the other hand, compared to employees, merchants (AOR = 0.3, 95% CI: 0.1–0.9), farmers and housewives (AOR = 0.3, 95% CI: 0.1–0.8), and participants categorized as “others” (AOR = 0.2, 95% CI: 0.1–0.7) had lower odds of possessing good knowledge of stroke risk factors. Although these occupational groups showed lower odds of good knowledge, the CIs, particularly for merchants, approach 1.0, suggesting limited precision. These findings should therefore be interpreted with caution.

Participants with a family history of stroke were 5.3 times more likely to have good knowledge compared to participants with no family history of stroke (2.1 and 13.0). Participants with appropriate follow‐up were 3.4 times more likely to have good knowledge compared to participants with no appropriate follow‐up (1.4 and 8.4). Participants who were informed by healthcare providers about stroke were 2.2 times more likely to have good knowledge compared to participants who were not informed (1.0 and 4.5) (Table 5).

**Table 5:** Bivariable and multivariable analysis for knowledge of risk factors and associated factors, prevention strategies, and treatment awareness among chronic disease patients in southern Ethiopia.

		Knowledge		Bivariate logistic regression		Multivariate logistic regression		
		Poor	Good	COR	p value	AOR	95% CI	
		N	N					
Type of institution	Governmental	295	86					
	Private	27	22	2.8	**0.001**	**2.6**	**1.1**	**5.9**
Age	18–40	83	21					
	41–60	164	66	1.6	**0.10**	1.8	0.8	4
	> 60	75	21	1.1	0.77	0.9	0.3	2.6
Sex	Female	167	44					
	Male	155	64	1.6	**0.046**	1.6	0.8	2.9
Educational status	No formal education	146	16					
	Grades 1–8	133	24	1.6	**0.15**	1.0	0.4	2.4
	Diploma	31	31	9.1	**≤ 0.001**	2.9	0.9	8.7
	Degree and above	12	37	28.1	**≤ 0.001**	6.5	**1.9**	**21.9**
Residence	Rural	164	97					
	Urban	158	11	8.5	**≤ 0.001**	3.3	**1.4**	**7.9**
Occupation	Employee	21	56					
	Merchant	80	22	0.1	**≤ 0.001**	**0.3**	**0.1**	**0.9**
	Farmer and housewife	194	22	0.04	**≤ 0.001**	**0.3**	**0.1**	**0.8**
	Others (students and retirees)	27	8	0.1	**≤ 0.001**	**0.2**	**0.1**	**0.7**
Duration of follow‐up	< 4years	187	52					
	≥ 4years	135	56	1.7	**0.07**	1.8	0.9	3.5
Hypertension	No	116	30					
	Yes	206	78	1.5	**0.1**	1.0	0.5	2.1
Family history	No	306	88					
	Yes	16	20	**4.35**	**≤ 0.001**	**5.3**	**2.1**	**13.0**
Appropriate follow‐up	No	103	10					
	Yes	219	98	**4.6**	**≤ 0.001**	**3.4**	**1.4**	**8.4**
HCP told	No	271	68					
	Yes	51	40	**3.1**	**≤ 0.001**	**2.2**	**1.0**	**4.5**

*Note:* The bold formatting reflects statistically significant associations based on this criterion.

It is noted that some independent variables, particularly those with smaller sample sizes in the reference or exposure categories, have wider 95% CIs. While the AORs indicate a statistically significant association, the width of the CIs reflects the increased sampling variability associated with these smaller subgroups, suggesting caution in generalizing the magnitude of these specific effect estimates to the broader population. The variables with wide CIs were retained as they demonstrated strong clinical and statistical significance, and the overall model diagnostics were sound.

### 3.6. Prevention Methods

Participants were asked about 10 stroke prevention strategies, selected based on current evidence‐based stroke prevention guidelines, recommendations from the Ethiopian Ministry of Health, and relevance to the chronic disease population in southern Ethiopia. Each correct response was scored as 1 and incorrect as 0. While all items were weighted equally for simplicity in scoring, we acknowledge that some strategies, such as blood pressure control and regular exercise, may have a larger effect on stroke risk than others. Future studies may consider differential weighting to reflect relative impact. Overall, only 33.3% of participants answered more than 50% of the prevention‐related questions correctly (Figure 1).

While a majority of participants were aware of the importance of regular physical exercise (39.8%) and appropriate hypertension treatment (36.5%), fewer recognized the benefits of avoiding or quitting smoking (30.2%), eating fruits and vegetables regularly (27.7%), and losing weight (37.4%).

Furthermore, knowledge of prevention methods for females, such as avoiding oral contraceptives and controlling blood sugar levels, was relatively low. Similarly, awareness of the importance of reducing fatty food consumption and avoiding excessive alcohol intake was limited (Table 6).

**Table 6:** Knowledge about stroke prevention methods among chronic disease patients in southern Ethiopia.

Question	Response			
	Correct		Incorrect	
	%value	95% CI	%value	95% CI
Engaging in regular physical exercise helps prevent stroke	39.8	35.1, 44.4	60.2	55.6, 64.8
Ensuring appropriate treatment of hypertension helps prevent stroke	36.5	31.6, 40.9	63.5	59.1, 68.4
Avoiding or quitting smoking helps prevent stroke	30.2	25.8, 34.7	69.8	65.3, 74.2
Eating fruits and vegetables regularly helps prevent stroke	27.7	23.3, 32.1	72.3	67.9, 76.7
Females should avoid the use of oral contraceptives to help prevent stroke	21.9	17.9, 25.8	78.1	74.2, 82.1
Losing weight (if overweight or obese) helps prevent stroke	37.4	33, 42.3	62.6	57.7, 67
Controlling blood sugar levels helps prevent stroke	34.7	30, 39.1	65.3	60.9, 70
Reducing the consumption of fatty foods helps prevent stroke	28.8	24.7, 33	71.2	67, 75.3
Avoiding excessive alcohol intake helps prevent stroke	27	23, 31.2	73	68.8, 77
Ensuring appropriate treatment of heart diseases helps prevent stroke	25.1	21.4, 29.3	74.9	70.7, 78.6

The mean score was 3.09 (SD = 2.01), with a similar distributional pattern. Observed scores range from 0 to 10. Scores did not cluster narrowly around the 50% cutoff, suggesting that the binary classification did not artificially inflate or distort prevalence estimates.

In summary, only 33.3% CI (29–38) of the participants correctly answered greater than 50% of the questions (Figure 1).

### 3.7. Awareness of Treatment Options for Stroke

Treatment awareness was evaluated using a two‐step approach, combining open‐ended and recognition‐based questions. For the open‐ended item, participants were asked to spontaneously describe what actions they would take in response to a stroke. Responses were coded independently by two reviewers using a predefined framework aligned with the recognition‐based options. Specifically, answers were categorized into the following domains: (1) medical management (blood pressure control; antiplatelet agents, e.g., aspirin; surgery; emergency hospital care; oil massage; and physiotherapy), (2) herbal/traditional remedies (local herbs), (3) religious/faith‐based practices (prayer, faith healing, and witchcraft), and (4) “Do not know.” Discrepancies in open‐ended coding were resolved through discussion until consensus was achieved. Interrater reliability was high, with a Cohen′s kappa of 0.84. A significant portion (62.6%) was unable to provide a correct answer. Among those who did respond, controlling blood pressure was the most commonly mentioned treatment option, followed by blood‐thinning agents, surgery, and traditional methods such as oil massage, faith healing, and witchcraft (Table 7).

**Table 7:** Stroke treatment awareness among chronic disease patients in southern Ethiopia.

Question	Response	*N*	%
How will a stroke be treated?	Do not know	269	62.6
	Control BP	150	34.9
	Oil massage/physiotherapy	15	3.5
	Blood‐thinning agents like aspirin	71	16.5
	Surgery	38	8.8
	Using local herbals like shiferaw	16	3.7
	Faith healing	29	6.7
	Magician/witchcraft	29	6.7

## 4. Discussion

This study identified markedly low levels of knowledge of stroke risk factors, prevention methods, and treatment awareness among chronic disease patients in southern Ethiopia. Moreover, the study identified several independent factors associated with participants′ knowledge about stroke risk factors. This finding aligns with recent studies demonstrating insufficient population knowledge of stroke symptoms and risk factors in Ethiopia and similar settings, where a substantial proportion of participants exhibited inadequate understanding of stroke [22, 23].

Importantly, the persistence of low knowledge among individuals with established chronic illnesses suggests that high clinical risk does not automatically translate into heightened awareness. From a health belief model (HBM) perspective, this may reflect low perceived susceptibility despite objective risk. Patients living with hypertension or diabetes may normalize their condition as manageable and therefore underestimate its potential progression to stroke. When perceived susceptibility remains low, individuals may not actively seek information or internalize preventive messages, even during repeated clinical encounters.

The HBM further posits that knowledge functions primarily as a cue to action rather than a determinant of behavior in itself. If perceived barriers, such as financial constraints, limited access to healthy foods, cultural norms, or fatalistic beliefs, outweigh perceived benefits, knowledge acquisition may remain limited or fail to motivate engagement with educational opportunities. This may partly explain why awareness of stroke risk factors and preventive behaviors remains insufficient even among patients in routine follow‐up care. Health behavior literature consistently demonstrates that the pathway from information to preventive action is mediated by perceived severity, perceived benefits, perceived barriers, and self‐efficacy [24, 25].

In our study, fewer than half of the participants recognized major stroke risk factors such as hypertension, diabetes, or heart disease, patterns observed in other community and hospital‐based studies [22, 26]. Notably, limited awareness persisted even among hypertensive patients, suggesting that routine chronic disease management may emphasize medication adherence while inadequately reinforcing the link between these conditions and stroke risk. From an HBM standpoint, clinical counseling may not sufficiently strengthen perceived severity or explicitly connect chronic illness to stroke outcomes.

The most frequently recognized risk factors were hypertension, DM, and heart disease. These risk factors were consistently recognized across various studies [27], including those conducted in Ethiopia, Sudan, Nigeria, and Pakistan [15, 28–30]. The strong association between these factors and stroke is well established in the medical literature [31, 32]. However, awareness of lifestyle‐related risks, including unhealthy diet, smoking, obesity, and excessive alcohol consumption, was notably lower. These behavioral factors require higher levels of perceived control and self‐efficacy to modify. According to the HBM, individuals may acknowledge biomedical risks more readily than lifestyle risks because behavioral change demands sustained effort and may be constrained by environmental and socioeconomic barriers [33, 34].

Only 25.1% of participants scored above 50% correct answers from the overall stroke risk factor question. The finding was lower compared to the findings of the study conducted in Ethiopia by [30] and in Nigeria by [15]. The discrepancy may be due to the study setting; previous study was only in hospital [30], whereas this study addresses all healthcare settings, which may lead to have participant population difference, in which the number of participants from rural residence who are uneducated and have limited exposure to information about stroke will be high—all of which may lower the knowledge of stroke risk factors [35]. The other reason for the discrepancy may be the difference between study populations [15, 30]. The participants in the previous studies were either hypertensive or had cardiac conditions, whereas our study included all chronic disease patients. These studies show that being hypertensive is positively associated with knowledge of stroke risk factors [21].

One of the methodological considerations in this study is the potential for selection bias arising from the recruitment process. Participants were recruited during routine follow‐up visits at chronic disease clinics, meaning the study population consisted entirely of patients already engaged in the healthcare system and adherent to their appointment schedules. In the context of health behavior literature, these “adherent” patients typically demonstrate higher levels of health literacy and engagement compared to those who are lost to follow‐up or do not seek regular care.

Therefore, the finding that only 25.1% of patients demonstrated “good knowledge” should be interpreted as a conservative estimate of stroke literacy among chronic disease patients in southern Ethiopia. If those already in regular contact with healthcare providers exhibit such marked knowledge deficits, the prevalence of stroke literacy in the broader, nonadherent chronic disease population in southern Ethiopia is likely even lower and more alarming. This suggests that the information gap is likely a systemic issue that extends beyond the clinic setting, necessitating community‐based outreach in addition to facility‐based education to reach those with the highest vulnerability.

Several sociodemographic factors were significantly associated with better knowledge of stroke risk factors, including higher educational attainment, urban residence, employment status, private healthcare use, family history of stroke, regular follow‐up, and receiving information from healthcare providers. Similar associations have been reported in multicountry and regional studies, where education level, urban location, and exposure to health information correlated with greater knowledge of stroke risk factors [36]. Participants who received services at private institutions were significantly more likely to have good knowledge compared to those served at governmental hospitals. Similar findings were reported from the studies conducted in Australia [37], Turkey [38], and South Africa [39]. Research suggests that patients attending private healthcare settings tend to have higher socioeconomic status and education levels compared to those in public facilities [40, 41], and participants with higher socioeconomic status and education levels have good knowledge of stroke risk factors [42, 43].

While participants receiving care in private healthcare facilities demonstrated higher stroke‐related knowledge, the policy interpretation of this association remains uncertain. Although the relationship persisted after adjustment for measured socioeconomic indicators (education, occupation, and residence), residual confounding cannot be excluded because direct measures of income or wealth were not available. Private facility utilization may therefore function partly as a proxy for unmeasured socioeconomic advantage rather than representing a true independent facility‐level effect.

Given the cross‐sectional design and absence of comprehensive socioeconomic measures, causal inference is limited. Future research incorporating detailed income data, qualitative assessments of care processes, and longitudinal designs would help disentangle facility‐level effects from socioeconomic selection and thereby better inform policy prioritization.

Individuals with higher educational levels were also more likely to possess good knowledge than those with no formal education. Consistent findings were reported from the studies conducted in Nigeria [43], Saudi Arabia [44], and Ethiopia [23]. Beyond improving literacy, education may enhance perceived susceptibility and perceived benefits of prevention, as educated individuals are more likely to process health information critically and relate it to personal risk. Education may also strengthen self‐efficacy, enabling individuals to feel capable of adopting preventive behaviors [45].

Additionally, urban residents demonstrated a higher likelihood of having good knowledge compared to rural residents. Supporting findings were reported from the studies conducted in Ethiopia [21, 30]. The observed urban–rural disparity in stroke knowledge can be multifaceted, as framed by the HBM. Urban environments may provide stronger cues to action through media exposure, community programs, and more frequent interaction with healthcare systems. In contrast, rural populations may experience structural barriers, including limited facility access and weaker information networks, which limit exposure to health messages. These structural barriers are compounded by diminished perceived behavioral control, stemming from restricted access to healthcare facilities and specialized providers in rural areas. Consequently, the lack of proximity to medical infrastructure not only limits direct health education but also reinforces the perception that stroke prevention and management are less attainable [21–23, 30]. Thus, urban–rural disparities likely reflect differences in informational access as well as variations in perceived barriers. And contradicting findings were reported from a study conducted in Mexico [46]. This discrepancy could arise from differences in curriculum focus or access to health education resources.

Regarding occupation, employees were more likely to have good knowledge than merchants, farmers, housewives, and others (students and retired). Similar findings were reported from a study conducted in Saudi Arabia [47]. Employees may benefit from structured environments that enhance exposure to health information and strengthen perceived control through financial stability and social support. Workplace settings may function as additional cues to action, facilitating health screening and education [48]. However, some estimates showed modest precision, and findings should be interpreted cautiously. Participants with a family history of stroke demonstrated better knowledge. This finding was supported by the findings from a study conducted in Ethiopia [30]. This might be potentially due to personal exposure and information‐seeking behaviors. However, increased knowledge does not necessarily translate into preventive action without addressing behavioral determinants, a consideration reinforced by health behavior research [33]. Individuals with familial exposure often demonstrate greater awareness yet may fail to implement sustained risk‐reducing actions. Distinguishing between knowledge acquisition and behavioral change is therefore critical.

Appropriate follow‐up and being informed by healthcare providers were also found to be positively associated with knowledge about stroke risk factors. Reports from other studies also support this finding [49]. Repeated clinical encounters may act as sustained cues to action and opportunities to reinforce perceived severity and benefits of prevention [50]. This underscores the central role of provider communication in shaping patient beliefs and knowledge.

Knowledge of stroke prevention methods and treatment options was also limited in this study. Only a minority correctly answered questions related to prevention, and most participants lacked awareness of evidence‐based treatment strategies beyond blood pressure control. This gap may contribute to delays in care‐seeking and suboptimal engagement in preventive practices.

Only 33.3% correctly answered more than half of the prevention‐related questions. The finding was higher compared to the study conducted in northern Ethiopia [16]. The discrepancy might be attributed to differences in population characteristics, study settings, and regional variations. The focus on chronic disease patients and the diverse healthcare settings in our study may have influenced knowledge levels, compared to the more homogeneous population and single‐hospital setting in the previous study. While a majority were aware of the importance of regular physical exercise and hypertension control, fewer recognized the benefits of avoiding smoking, eating a healthy diet, and losing weight. Additionally, knowledge of prevention methods specific to females, such as avoiding oral contraceptives and controlling blood sugar levels, was particularly low. These findings underscore the urgent need for improved stroke awareness and prevention strategies among chronic disease patients. Healthcare providers should focus on enhancing patient education, especially for vulnerable groups, to reduce the risk of stroke and its complications [16, 51].

Over 60% of participants were unable to provide a correct answer, and among those who did, controlling blood pressure was the most frequently mentioned treatment option. This indicates a need for improved education on evidence‐based stroke care, including the role of blood‐thinning agents, surgery, and other effective treatments.

### 4.1. Conclusion and Recommendation

This study demonstrates critically low knowledge of stroke risk factors, prevention strategies, and treatment options among high‐risk chronic disease patients in southern Ethiopia. Guided by the HBM, the findings suggest that deficits in perceived susceptibility, perceived benefits, self‐efficacy, and exposure to effective cues to action likely contribute to persistent knowledge gaps.

While this study highlights significant deficits in stroke literacy among chronic disease patients in southern Ethiopia, it is recognized that knowledge acquisition alone is often insufficient to drive direct preventive action. However, within the framework of health behavior change, documenting these knowledge deficits represents a critical first step in a broader, multicomponent prevention strategy. Stroke‐related knowledge serves as a foundational prerequisite for informed health decision‐making and may enhance the effectiveness of established HBM constructs such as perceived susceptibility, perceived severity, and cues to action, providing the foundational awareness required for patients to engage with more complex behavioral interventions.

In the context of southern Ethiopia′s healthcare landscape, improving stroke literacy should not be viewed as an isolated goal, but rather as an essential pillar of a comprehensive public health approach. This pillar must function alongside strengthened clinical management of chronic conditions and structural health system improvements to effectively reduce stroke incidence. By addressing the “information gap” identified in this study, healthcare programs can better prepare high‐risk patients to overcome perceived barriers and enhance their self‐efficacy for long‐term stroke prevention.

Interventions should therefore move beyond information dissemination alone. Integrating structured stroke education into routine chronic disease follow‐up, strengthening provider communication skills, enhancing community‐based outreach in rural areas, and leveraging media as cues to action are essential. Educational strategies should explicitly link chronic diseases to stroke risk to strengthen perceived susceptibility, address structural and cultural barriers, and promote self‐efficacy. Multifaceted interventions targeting both cognitive and structural determinants are necessary to translate awareness into sustained preventive behavior.

### 4.2. Limitations and Future Directions

Participants were recruited during routine follow‐up visits, potentially underrepresenting nonadherent patients and introducing selection bias toward individuals more engaged in care, which may have inflated knowledge estimates. The cross‐sectional design precludes causal inference, and the focus on a single population in southern Ethiopia limits generalizability. The use of an arbitrary ≥ 50% cutoff to define “good knowledge” may have led to information loss through dichotomization and does not necessarily reflect clinically meaningful adequacy, despite facilitating comparison with prior studies. In addition, stroke prevention knowledge was assessed using an unweighted scoring system that treated all risk factors equally, reflecting general awareness rather than clinical prioritization of high‐impact factors. Residual confounding from unmeasured socioeconomic factors cannot be excluded, particularly when interpreting differences between private and public health facilities. Reliance on self‐reported, recognition‐based questions may also have introduced recall and social desirability biases. Educational sessions were intentionally withheld during data collection to avoid contamination, which may represent an ethical limitation. Future research should employ longitudinal designs, include broader populations, develop empirically grounded and potentially weighted knowledge indices, and evaluate the long‐term impact of targeted educational interventions on stroke‐related outcomes.

## Funding

This study was funded by Dilla University, 10.13039/501100021788.

## Conflicts of Interest

The authors declare no conflicts of interest.

## Data Availability

The datasets used in this study are available from the corresponding author on reasonable request.
